# Clinicopathological Characteristics and Prognosis of IgA Nephropathy Combined With Malignant Hypertension Kidney Injury

**DOI:** 10.1002/jcla.70105

**Published:** 2025-09-19

**Authors:** Zhong Zhong, Wenzhuo Yang, Zefang Dai, Jianbo Li, Zhijian Li

**Affiliations:** ^1^ Department of Nephrology The First Affiliated Hospital, Sun Yat‐Sen University Guangzhou China; ^2^ NHC Key Laboratory of Clinical Nephrology (Sun Yat‐Sen University) and Guangdong Provincial Key Laboratory of Nephrology Guangzhou China; ^3^ Department of Medical Oncology Beijing Hospital, National Center of Gerontology, Institute of Geriatric Medicine, Chinese Academy of Medical Sciences Beijing China

**Keywords:** cohort study, IgA nephropathy, malignant hypertension kidney injury, renal outcome

## Abstract

**Background:**

IgA nephropathy (IgAN), a major cause of primary glomerulonephritis, is closely associated with malignant hypertension (MHT). This study aimed to explore the clinicopathological characteristics and renal prognosis of patients with IgAN combined with malignant hypertension kidney injury (IgAN‐MHT) and investigate the associations of clinicopathological indicators with prognosis.

**Methods:**

In this single‐center retrospective cohort study, patients diagnosed with IgAN‐MHT through kidney biopsy between January 1, 2008, and June 30, 2023, in our hospital were enrolled. Cox regression models were used to assess the associations between clinical indicators and renal prognosis in IgAN‐MHT patients.

**Results:**

A total of 70 patients were included in the analysis of renal function improvement. During a median follow‐up time of 9.4 months, 26 patients (37.1%) achieved renal function improvement. Patients in the improved renal function group had higher hemoglobin levels (*p* = 0.003), lower serum creatinine levels (*p* = 0.006), a higher proportion of patients using sulodexide (*p* = 0.018), and a lower proportion of glomerulosclerosis (*p* < 0.001). Multivariable Cox regression analysis indicated that a higher proportion of glomerulosclerosis was independently associated with a lower likelihood of renal function improvement (HR = 0.97, 95% CI = 0.96–0.99, *p* = 0.003), while sulodexide administration was independently associated with a higher likelihood of improvement (HR = 2.75, 95% CI = 1.18–6.41, *p* = 0.019).

**Conclusion:**

This study revealed that in IgAN‐MHT patients, a higher proportion of glomerulosclerosis was independently associated with poorer renal outcomes, while the use of sulodexide showed a significant independent association with improved renal function.

## Graphical abstract

Kaplan–Meier cumulative event incidence analysis for improved renal function in IgAN‐MHT patients. The cumulative rates of improved renal function in IgAN‐MHT patients at 1, 2, 3, 4, and 5 years were 31.1%, 38.8%, 42.2%, 42.2%, and 52.6%, respectively.

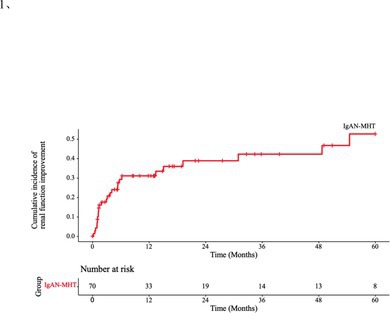

## 1 Introduction

Malignant hypertension (MHT) is an acute hypertensive crisis, which is marked by a sudden increase in blood pressure and acute organ injury [1]. MHT‐related renal damage refers to the pathological injury to renal tissue caused by MHT [2]. Clinical manifestations of MHT kidney injury often include elevated serum creatinine levels and mild to moderate proteinuria, while typical pathological changes often resemble thrombotic microangiopathy (TMA)‐like changes [3, 4].

IgA nephropathy (IgAN) is the most prevalent type of primary glomerulonephritis worldwide [5] and is characterized by the deposition of IgA‐containing immune complexes in the mesangial area of glomeruli and histopathological lesions of mesangial cell proliferation [6, 7]. The most common clinical manifestation of IgAN is asymptomatic hematuria, which is often accompanied by varying degrees of proteinuria, with or without impaired renal function [8]. The clinical and pathological characteristics of IgAN vary widely among patients, with 20% to 50% of patients progressing to end‐stage kidney disease (ESKD) within 20 years after disease onset [9, 10].

IgAN combined with MHT kidney injury (IgAN‐MHT) refers to the coexistence of pathological features of both IgAN and MHT, as confirmed by kidney biopsy. IgAN is a major cause of secondary MHT, accounting for approximately 9% of the causes of MHT [11]. Concurrently, MHT represents a specific clinical manifestation of IgAN. In patients with IgAN, the incidence of thrombotic microangiopathy (TMA) increases with rising blood pressure and is associated with poorer renal outcomes [12]. Recently, we have found that among patients with MHT renal injury confirmed by renal biopsy, 29.5% had concurrent IgAN [13]. Previous studies have indicated that the renal prognosis for patients with IgAN‐MHT is poor and often progresses to ESKD within a short period, with no specific effective treatment available [14, 15]. Renal pathology may offer prognostic value in this population. Chen et al. reported that mesangial proliferation in glomeruli is an independent risk factor for renal prognosis in IgAN patients with MHT [16]. However, current observational clinical studies in IgAN‐MHT patients remain limited by small sample sizes, low diagnostic rates, and inadequate follow‐up.

In this study, 70 patients with IgAN‐MHT who were followed regularly for over a decade were enrolled. We aimed to explore the clinical and pathological characteristics of IgAN‐MHT patients, analyze the likelihood of improved renal function in IgAN‐MHT patients, and identify the factors influencing improved renal function.

## 2 Methods

### 2.1 Study Subjects

This was a single‐center cohort study that enrolled patients from the Department of Nephrology, First Affiliated Hospital of Sun Yat‐sen University between January 1, 2008, and June 30, 2023. The eligibility criteria were: (1) diagnosed with MHT kidney injury through kidney biopsy; (2) pathological findings consistent with IgAN; and (3) age ≥ 18 years at the time of diagnosis. The exclusion criteria were: (1) renal biopsy indicating either the absence of renal parenchymal disease or the presence of non‐IgAN‐related kidney diseases; (2) normal baseline serum creatinine levels (Since the purpose of this study was to explore the factors influencing the improvement of renal function in patients with IgAN‐MHT); (3) incomplete baseline data or missing follow‐up information on renal function; and (4) a follow‐up duration of less than 3 months.

The diagnosis of MHT was based on the detection of a hypertensive emergency characterized by the presence of severe BP elevation (usually a diastolic BP levels over 120 mmHg), and accompanied by Grade III or IV hypertensive retinopathy according to the Keith–Wagener–Barker classification and/or evidence of imminent or progressive target organ dysfunction secondary to hypertension [17, 18]. The diagnosis of MHT kidney injury was confirmed based on renal pathological features, including various pathological changes such as capillary loop wrinkling, capsule thickening, significant renal artery intimal thickening, vessel wall “onion‐peel” thickening, fibrinoid necrosis, intravascular thrombosis, ischemic glomerular alterations, and tubular necrosis [4, 19].

### 2.2 Study Endpoint

The primary endpoint of this study was improved renal function. On the basis of previous definitions of improved renal function [18, 20], this study defined improved renal function as meeting any of the following criteria, sustained for at least 1 month: (1) for patients not requiring dialysis prior to and during hospitalization for renal biopsy, a decrease in the serum creatinine level of more than 25% from the baseline peak creatinine level; (2) for patients not requiring dialysis prior to and during hospitalization for renal biopsy, a decrease in the serum creatinine level to normal levels; (3) for patients requiring dialysis prior to and during hospitalization for renal biopsy, achieving dialysis discontinuation. Meeting any one of these criteria was considered to indicate achievement of the primary endpoint. The follow‐up end date of this study is June 30, 2023.

### 2.3 Definition of Baseline Peak Creatinine Level

The baseline peak creatinine level was defined as follows: (1) for patients not requiring dialysis prior to and during hospitalization for renal biopsy, the highest serum creatinine level during hospitalization and within the 3 months preceding hospitalization; or (2) for patients requiring dialysis prior to and during hospitalization for renal biopsy, the highest serum creatinine level within the 3 months prior to dialysis.

### 2.4 Data Collection

Baseline data were collected during hospitalization for renal biopsy and included sociodemographic information (gender, age, and dialysis status), clinical characteristics (height, weight, and body mass index), serum laboratory parameters (levels of hemoglobin, platelet, serum creatinine, uric acid, albumin, triglycerides, low‐density lipoprotein cholesterol), 24‐h urine protein quantification, retinopathy grading, renal pathology (status of glomeruli, tubules, interstitium, and vasculature), and long‐term medication use (angiotensin‐converting enzyme inhibitors (ACEI), angiotensin receptor blockers (ARB), angiotensin receptor/neprilysin inhibitors (ARNI), statins, beraprost sodium, and sulodexide). Serum creatinine levels during follow‐up were obtained from medical records at our center or other institutions, and changes from the baseline peak creatinine level were calculated. For patients who were dialysis‐dependent at baseline, dialysis discontinuation status during follow‐up was also recorded.

## 3 Statistical Analysis

Baseline data from the study subjects were compared between the improved renal function group and the nonimprovement group among patients with IgAN‐MHT. For continuous quantitative data, normality tests were performed. Normally distributed data were expressed as the means ± standard deviations and were compared via Student's *t*‐test. Nonnormally distributed data were expressed as medians (interquartile ranges) and were compared via Mann–Whitney *U* test. Categorical variables are expressed as percentages (%). Unordered categorical variables were compared via chi‐squared test or Fisher's exact test, whereas ordered categorical variables were compared via rank‐sum test. Kaplan–Meier cumulative event incidence analysis was performed to assess improved renal function in patients with IgAN‐MHT, and the cumulative rates of improved renal function at 1, 2, 3, 4, and 5 years were calculated. Univariable Cox proportional hazards regression models were used to explore the influence of covariates on the endpoint event, and covariates with *p* values less than 0.05 in the univariable analysis were included in the multivariable Cox regression analysis to investigate the independent effects of covariates on the endpoint event. A two‐sided *p* value less than 0.05 was considered to indicate statistical significance, and all the statistical analyses were conducted via R software (version 4.3.1).

## 4 Results

### 4.1 Characteristics of the Study Subjects

A total of 306 patients diagnosed with MHT kidney injury in our hospital from January 1, 2008, to June 30, 2023, were included in this study. Among these patients, 217 patients with MHT kidney injury but without coexisting IgAN, 2 patients with normal baseline serum creatinine levels, 3 patients with follow‐up data of less than 3 months, and 14 patients lacking follow‐up data concerning renal function were excluded. Eventually, 70 patients were included for analysis. The patients were grouped on the basis of whether they achieved improved renal function. Twenty‐six patients were in the improved renal function group, and 44 patients were in the nonimprovement group.

### 4.2 Baseline Clinical and Pathological Characteristics

The baseline clinical and pathological characteristics of the IgAN‐MHT improved renal function group and nonimprovement group are presented in Table 1 and Table 2. In terms of baseline clinical characteristics, compared with patients in the nonimprovement group, patients in the improved renal function group had significantly higher hemoglobin levels (*p* = 0.003), lower serum creatinine levels (*p* = 0.006), and a higher proportion of patients administered sulodexide (*p* = 0.018). In terms of baseline pathological characteristics, patients in the improved renal function group had a significantly lower proportion of glomerulosclerosis compared with those in the nonimprovement group (*p* < 0.001).

**TABLE 1:** Clinical characteristics of IgAN‐MHT patients.

Clinical characteristics	Total (*n* = 70)	Nonimprovement group (*n* = 44)	Improvement group (*n* = 26)	*p* ^a^
Male (*n*, %)	59 (84.3)	38 (86.4)	21 (80.8)	0.778
Age (year)	30 (27, 36)	30 (26, 37)	32 (28, 35)	0.503
BMI (kg/m^2^)	23.2 ± 2.9	23.4 ± 2.9	22.8 ± 3.0	0.422
Hb (g/L)	96 (80, 116)	88 (76, 109)	110 (93, 122)	0.003
PLT (×10^9^/L)	248 ± 67	243 ± 73	257 ± 57	0.417
Scr (μmol/L)	646 (397, 973)	800 (475, 1274)	494 (373, 660)	0.006
eGFR (mL/min/1.73 m^2^)	9.3 (5.7, 16.9)	7.5 (4.2, 13.7)	12.5 (8.8, 18.7)	0.006
UA (μmol/L)	494 ± 135	506 ± 148	475 ± 108	0.353
LDL‐c (mmol/L)	3.0 ± 1.1	2.9 ± 1.0	3.1 ± 1.2	0.578
TG (mmol/L)	1.6 (1.3, 2.3)	1.8 (1.3, 2.3)	1.5 (1.3, 2.4)	0.572
ALB (g/L)	33.8 (31.3, 38.7)	33.8 (31.2, 37.4)	34.2 (31.4, 38.9)	0.597
Urine protein quantification (g/24 h)	2.4 (1.5, 3.2)	2.5 (1.5, 3.2)	2.2 (1.5, 3.1)	0.473
Retinopathy (stage 4) (*n*, %)	17 (24.3)	12 (27.3)	5 (19.2)	0.639
ACEI and/or ARB (*n*, %)	40 (57.1)	21 (47.7)	19 (73.1)	0.069
ARNI (*n*, %)	22 (31.4)	16 (36.4)	6 (23.1)	0.373
Statins (*n*, %)	31 (44.3)	17 (38.6)	14 (53.8)	0.323
Sulodexide (*n*, %)	29 (41.4)	13 (29.5)	16 (61.5)	0.018
Beraprost sodium (*n*, %)	9 (12.9)	3 (6.8)	6 (23.1)	0.111

*Note:* Values are expressed as mean ± standard, median (interquartile range), or number (%).

Abbreviations: ACEI, angiotensin‐converting enzyme inhibitors; ALB, albumin; ARB, angiotensin receptor blockers; ARNI, angiotensin receptor/neprilysin inhibitors; BMI, body mass index; Hb, hemoglobin; IgAN‐MHT, IgA nephropathy combined with malignant hypertension kidney injury; LDL‐c, low‐density lipoprotein cholesterol; PLT, Platelet; Scr, serum creatinine; TG, triglyceride; UA, urine acid.

^**a**^ *p* < 0.05 is considered statistically significant.

**TABLE 2:** Pathological characteristics of IgAN–MHT.

Pathological characteristics	Total (*n* = 70)	Nonimprovement (*n* = 44)	Improvement (*n* = 26)	*p* ^a^
Glomerulosclerosis proportion (%)	66.7 (47.9, 85.4)	76.3 (61.7, 88.0)	43.9 (25.9, 72.9)	< 0.001
Segmental glomerulosclerosis proportion (%)	1.1 (0, 7.6)	0 (0, 7.9)	3.2 (0, 6.6)	0.922
Degree of tubular atrophy/interstitial fibrosis				0.669
< 25% (*n*, %)	0	0	0	
25%–50% (*n*, %)	13 (18.6)	7 (15.9)	6 (23.1)	
> 50% (*n*, %)	57 (81.4)	37 (84.1)	20 (76.9)	
Detached brush margin of tubular epithelial cells/flattened cells (*n*, %)	11 (15.7)	7 (15.9)	4 (15.4)	1.000
Vascular fibrinoid necrosis (*n*, %)	22 (31.4)	15 (34.1)	7 (26.9)	0.721
Vascular onion skin changes (*n*, %)	38 (54.3)	22 (50.0)	16 (61.5)	0.491
Intravascular thrombosis (*n*, %)	13 (18.6)	7 (15.9)	6 (23.1)	0.669
Intravascular RBC fragments (*n*, %)	10 (14.3)	4 (9.1)	6 (23.1)	0.207

*Note:* Values are calculated as mean ± standard, median (interquartile range), or number (%). Degree of tubular atrophy/interstitial fibrosis grouping refers to the grading of tubular atrophy/interstitial fibrosis (T) in the Oxford classification of IgA nephropathy MEST‐C: T0 (< 25%), T1 (25%–50%), T2 (> 50%).

Abbreviations: IgAN–MHT, IgA nephropathy combined with malignant hypertension kidney injury; RBC, Red Blood Cells.

^**a**^ *p* < 0.05 is considered statistically significant.

### 4.3 Kaplan–Meier Cumulative Event Incidence Analysis for Improved Renal Function

**FIGURE 1:**
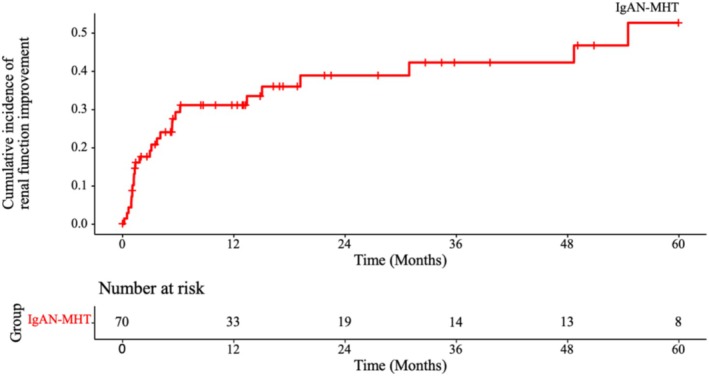
Kaplan–Meier cumulative event incidence analysis for improved renal function in IgAN‐MHT patients.

The median follow‐up duration was 9.4 months, during which 26 patients (37.1%) achieved improved renal function. The median time to improved renal function was 11.0 (3.1, 31.4) months. The Kaplan–Meier cumulative event incidence analysis for improved renal function in IgAN‐MHT patients is illustrated in Figure 1. The cumulative rates of improved renal function in IgAN‐MHT patients at 1, 2, 3, 4, and 5 years were 31.1%, 38.8%, 42.2%, 42.2%, and 52.6%, respectively (Table 3).

**TABLE 3:** Cumulative rates of improved renal function in IgAN‐MHT patients.

Group	Time (months)				
	12	24	36	48	60
IgAN‐MHT	31.1% (20.1%, 42.8%)	38.8% (25.7%, 51.8%)	42.2% (28.0%, 55.8%)	42.2% (28.0%, 55.8%)	52.6% (33.3%, 68.7%)

Abbreviations: IgAN‐MHT, IgA nephropathy combined with malignant hypertension kidney injury.

### 4.4 Univariable Cox Regression Analysis of Improved Renal Function

A univariable Cox regression model was constructed incorporating sociodemographic data, laboratory test results, retinal examination results, long‐term medication treatment, and renal pathological characteristics to analyze factors associated with the occurrence of improved renal function. The results revealed that higher hemoglobin levels (HR = 1.16, 95% CI = 1.02–1.32, *p* = 0.028) and the use of sulodexide (HR = 2.59, 95% CI = 1.17–5.71, *p* = 0.018) were associated with improved renal function in IgAN‐MHT patients. Conversely, higher serum creatinine levels (HR = 0.84, 95% CI = 0.75–0.95, *p* = 0.004) and glomerulosclerosis proportion (HR = 0.97, 95% CI = 0.95–0.98, *p* < 0.001) were associated with nonimproved renal function (Table 4).

**TABLE 4:** Univariable Cox regression analysis of improved renal function in IgAN‐MHT patients.

	HR	95% CI	*p* ^a^
Gender (male/female)	0.89	0.33 ~ 2.38	0.820
Age (per 1 year)	0.99	0.95 ~ 1.04	0.680
BMI (per 1 kg/m^2^)	0.97	0.85 ~ 1.10	0.621
Hb (per 10 g/L)	1.16	1.02 ~ 1.32	0.028
PLT (per 10 × 10^9^/L)	1.03	0.98 ~ 1.09	0.229
Scr (per 100 μmol/L)	0.84	0.75 ~ 0.95	0.004
UA (per 10 μmol/L)	0.99	0.96 ~ 1.02	0.448
LDL‐c (per 1 mmol/L)	1.28	0.86 ~ 1.90	0.217
TG (per 1 mmol/L)	0.76	0.46 ~ 1.26	0.289
ALB (per 1 g/L)	1.00	0.94 ~ 1.07	0.969
Urine protein quantification (per 1 g/24 h)	0.96	0.75 ~ 1.22	0.735
Retinopathy (stage 4)	0.89	0.34 ~ 2.37	0.819
ACEI and/or ARB (yes/no)	1.70	0.71 ~ 4.05	0.232
ARNI (yes/no)	0.85	0.34 ~ 2.14	0.733
Statins (yes/no)	1.63	0.75 ~ 3.55	0.215
Sulodexide (yes/no)	2.59	1.17 ~ 5.71	0.018
Beraprost sodium (yes/no)	2.02	0.80 ~ 5.07	0.136
Glomerulosclerosis proportion (per 1%)	0.97	0.95 ~ 0.98	< 0.001
Segmental glomerulosclerosis proportion (per 1%)	0.97	0.91 ~ 1.05	0.478
**Degree of tubular atrophy/interstitial fibrosis**			
T1	Ref.		
T2	0.62	0.25 ~ 1.55	0.302
Detached brush margin of tubular epithelial cells/flattened cells (yes/no)	1.01	0.35 ~ 2.95	0.978
Vascular fibrinoid necrosis (yes/no)	0.82	0.34 ~ 1.96	0.660
Vascular onion skin changes (yes/no)	1.15	0.52 ~ 2.56	0.732
Intravascular thrombosis (yes/no)	1.69	0.68 ~ 4.24	0.260
Intravascular RBC fragments (yes/no)	2.06	0.82 ~ 5.14	0.122

*Note:* Degree of tubular atrophy/interstitial fibrosis grouping refers to the grading of tubular atrophy/interstitial fibrosis (T) in the Oxford classification of IgA nephropathy MEST‐C: T0 (< 25%), T1 (25%–50%), T2 (> 50%). There was no grade T0 among the IgAN‐MHT patients included in this study.

Abbreviations: ACEI, angiotensin‐converting enzyme inhibitors; ALB, albumin; ARB, angiotensin receptor blockers; ARNI, angiotensin receptor/neprilysin inhibitors; BMI, body mass index; Hb, hemoglobin; LDL‐c, low‐density lipoprotein cholesterol; PLT, Platelet; Scr, serum creatinine; TG, triglyceride; UA, urine acid.

^**a**^ *p* < 0.05 is considered statistically significant.

### 4.5 Multivariable Cox Regression Analysis of Improved Renal Function

The aforementioned covariates associated with improved renal function in IgAN‐MHT patients were included in a multivariable Cox regression model (Table 5). The results indicated that in IgAN‐MHT patients, the administration of sulodexide was independently associated with improved renal function (HR = 2.75, 95% CI = 1.18–6.41, *p* = 0.019), whereas glomerulosclerosis proportion was independently associated with nonimproved renal function (HR = 0.97, 95% CI = 0.96–0.99, *p* = 0.003) (Table 5).

**TABLE 5:** Multivariable Cox regression analysis of improved renal function in IgAN‐MHT patients.

	HR	95% CI	*p* ^a^
Hemoglobin (per 10 g/L)	0.87	0.71–1.08	0.202
Serum creatinine (per 100 μmol/L)	0.85	0.71–1.02	0.080
Sulodexide (yes/no)	2.75	1.18–6.41	0.019
Glomerulosclerosis proportion (per 1%)	0.97	0.96–0.99	0.003

^**a**^ *p* < 0.05 is considered statistically significant.

## 5 Discussion

This study revealed that compared with those without improved renal function, IgAN‐MHT patients with improved renal function had higher hemoglobin and lower serum creatinine levels, a higher proportion of administration of sulodexide, and a lower proportion of glomerulosclerosis. The use of sulodexide was independently associated with improved renal function in IgAN‐MHT patients, whereas the proportion of glomerulosclerosis was associated with nonimproved renal function.

In the analysis of baseline clinical and pathological characteristics, this study revealed that IgAN‐MHT patients with improved renal function had higher hemoglobin levels. In contrast to the moderate anemia observed in patients without improved renal function, those in the improvement group exhibited only mild anemia. Additionally, univariable Cox regression analysis indicated that elevated hemoglobin levels were associated with improved renal function. Previous research noted anemia severity may be related to the progression of chronic kidney disease [21]. Furthermore, in the context of MHT, anemia may also result from hemoglobin consumption, indicating increased TMA severity and activation of the complement system [22]. However, the association between hemoglobin and renal prognosis was no longer significant in the multivariable analysis. This discrepancy may be related to several factors: First, other covariates in the multivariable Cox regression model may have explained part of the variation, thereby weakening the independent effect of hemoglobin on the prognosis of IgAN‐MHT patients. Second, the relatively limited sample size of this study may have restricted the statistical power, preventing the detection of the true effect of hemoglobin. Additionally, there may be other unadjusted confounding factors that affected the relationship between the variable hemoglobin and prognosis.

Serum creatinine levels are also critical indicators reflecting the severity of renal injury and the chronicity of kidney diseases. Research has shown that a baseline serum creatinine level of less than 300 μmol/L is an independent predictor of stable or improved renal function in patients with MHT [23]. Simultaneously, Amraoui et al. reported that a high serum creatinine level at onset is a risk factor for progression to ESKD in patients with MHT [24]. Our findings indicate that IgAN‐MHT patients with improved renal function had lower serum creatinine levels, suggesting that those with higher baseline serum creatinine levels faced greater challenges in achieving improved renal function.

This study revealed that the use of sulodexide was independently associated with improved renal function in IgAN‐MHT patients. Sulodexide is a polysaccharide antithrombotic agent commonly used clinically to improve urinary protein levels and protect renal function in patients with diabetic nephropathy [25, 26]. It has antithrombotic properties, promotes fibrinolysis, suppresses inflammatory responses, protects endothelial cells, and regulates vascular function [27]. TMA is a crucial pathological mechanism of kidney injury in MHT patients and is characterized by endothelial cell damage leading to microvascular thrombosis and ischemic injury to tissues and organs [28]. The multitarget pharmacological effects of sulodexide are directly related to the core pathological features of TMA. First, a recent study has shown that sulodexide can protect endothelial cells against 4‐hydroxynonenal‐induced oxidative stress and glutathione‐dependent redox imbalance by modulation of the sestrin2/nuclear factor erythroid 2‐related factor 2 pathway [29]. Second, sulodexide is a highly purified mixture of glycosaminoglycans composed of fast‐moving heparin and dermatan sulfate, which exerts its antithrombotic effects by binding to antithrombin and heparin cofactor II while promoting endothelial repair [30]. In addition, it has been found that the degradation products of the endothelial glycocalyx are increased in the serum of TMA patients, while the glycocalyx density is reduced in TMA patients and is significantly associated with local complement activation [31]. Sulodexide can replenish the endothelial glycocalyx [32] and thus provide corresponding protective effects in TMA.

Furthermore, the proportion of glomerulosclerosis serves as an indicator of the degree of sclerosis in the glomerular structure and is associated with irreversible loss of glomerular filtration function. Previous research from our center has shown that in IgAN patients, glomerulosclerosis severity progressively increases with disease duration, serving as an irreversible pathological indicator of poor renal prognosis [33]. Our findings demonstrated that the baseline proportion of glomerulosclerosis was significantly greater in the nonimprovement group than in the improved renal function group, and the increase in glomerulosclerosis proportion served as an independent risk factor for nonimproved renal function, which suggests that glomerulosclerosis proportion can be used as an indicator for judging the renal prognosis in IgAN‐MHT patients.

In this study, we explored the clinical and pathological characteristics associated with improvements in renal function, providing insights for the assessment of patient condition and methods for improving renal function for IgAN‐MHT patients in clinical practice. However, certain limitations in the present study should be recognized. First, this study lies in its single‐center observational study, which may introduce bias due to the regional population and disease characteristics, thus limiting the generalizability and applicability of the findings. Second, considering the small sample size and the relatively low number of endpoint events in this study, we did not forcibly include other potential prognostic factors for analysis, which may contribute to our results being partially biased by these factors. Finally, the results of this study cannot reveal causal correlations because of the observational design.

## 6 Conclusion

In conclusion, this study revealed that the occurrence of improved renal function in IgAN‐MHT patients is associated with certain baseline clinical and pathological characteristics at disease onset. Compared with patients without improved renal function, those with improved renal function had higher hemoglobin levels and lower serum creatinine levels. Additionally, a greater proportion of glomerulosclerosis in renal pathology was associated with greater difficulty in achieving improved renal function in IgAN‐MHT patients. In terms of treatment, we found that the administration of sulodexide was independently associated with improved renal function in IgAN‐MHT patients. Given its common clinical use as an antithrombotic agent, the use of sulodexide in IgAN‐MHT patients may improve disease prognosis, but its effectiveness and dosage‐associated guidance warrant further investigation in future studies. This research provides valuable reference data for the assessment and treatment of IgAN‐MHT patients.

## Author Contributions

Zhijian Li and Jianbo Li designed the study. Zhong Zhong, Zefang Dai, and Wenzhuo Yang collected the clinical data of patients and analyzed the data. Zhong Zhong and Wenzhuo Yang drafted the manuscript. Jianbo Li and Zhijian Li checked and revised the article.

## Ethics Statement

This study was carried out according to the ethical principles of the Declaration of Helsinki and was approved by the Research Ethics Committee of The First Affiliated Hospital, Sun Yat‐sen University (IRB approval number [2022] 710).

## Consent

All participants provided written informed consent.

## Data Availability

The data that support the findings of this study are available from the corresponding author upon reasonable request.
